# Extending Aging of Beef *Longissimus Lumborum* From 21 to 84 Days Postmortem Influences Consumer Eating Quality

**DOI:** 10.3390/foods9020208

**Published:** 2020-02-17

**Authors:** Andrea Garmyn, Nicholas Hardcastle, Rod Polkinghorne, Loni Lucherk, Mark Miller

**Affiliations:** 1 Department of Animal and Food Sciences, Texas Tech University, Lubbock, TX 79409, USA; nicholas.hardcastle@ttu.edu (N.H.); mfmrraider@aol.com (M.M.); 2 Birkenwood Pty. Ltd., 46 Church St, Hawthorn, VIC 3122, Australia; rod.polkinghorne@gmail.com; 3 Department of Agricultural Sciences, West Texas A&M University, Canyon, TX 79016, USA; llucherk@wtamu.edu

**Keywords:** beef, consumer, eating quality, extended postmortem aging, sensory testing

## Abstract

Our objective was to determine the effect of extending postmortem aging from 21 to 84 days on consumer eating quality of beef *longissimus lumborum*. Strip loins were collected from 108 carcasses. The *longissimus lumborum* muscle was isolated from strip loins and assigned to one of ten postmortem aging periods from 21 to 84 days (7-day increments) and balanced within four anatomical positions within the muscle. Consumer evaluations for tenderness, juiciness, flavor, and overall liking were conducted using untrained consumer sensory panels consisting of 1080 individual consumers, in accordance with the Meat Standards Australia protocols. These scores were then used to calculate an overall eating quality (MQ4) score. Postmortem aging had no effect (*P* > 0.05) on tenderness, but juiciness, flavor liking, overall liking, and MQ4 declined (*P* < 0.05) as aging period increased. Samples aged 21 to 42 days were most preferred having greater (*P* < 0.05) overall liking and greater (*P* < 0.05) MQ4 scores than samples aged 70 to 84 days postmortem. These results suggest that *longissimus lumborum* samples should not be wet-aged longer than 63 days to prevent potential negative eating experiences for consumers; however, altering storage conditions, specifically reducing temperature, could potentially allow for longer chilled storage without such negative effects on flavor and overall liking.

## 1. Introduction

The effects of postmortem aging of beef *longissimus* tenderness are relatively well documented [1,2] up to 28 days postmortem. However, the 2015 National Beef Tenderness Survey indicated average postfabrication storage or aging times for retail strip loin and foodservice top loin in the Unites States were 27.2 days and 34.6 days, respectively, but could range up to 101 days [3]. Moreover, the effects of longer term (>28 days) postmortem aging on consumer perception of eating quality are less well defined and more variable, especially in other palatability traits, such as flavor or juiciness.

Colle et al. [4] found increasing postmortem aging of *longissimus lumborum* steaks from 2 to 14 days improved tenderness, but no additional improvement was observed after 14 days. Also, consumer acceptability, juiciness, and flavor liking were similar from 2 through 63 days of postmortem aging [4]. Tenderness can be improved by aging strip loin subprimals for 56 days postmortem, but flavor intensity can decline with an increase in off-flavor intensity at this aging length [5]. However, Hughes et al. [6] reported an improvement in all consumer eating quality traits (tenderness, juiciness, flavor and overall liking) of beef *longissimus* from 2 to 12 weeks of postmortem aging, but no further improvement when extending postmortem aging to 20 weeks.

Given the potential variation in the postmortem aging length of beef, and especially *longissimus lumborum*, available at retail in the US, there is a need to better quantify the effect of extended postmortem aging on consumer eating quality of beef *longissimus lumborum*. To our knowledge, no studies have investigated extended aging of US beef *longissimus lumborum* through 12 weeks (84 days) of chilled vacuum-packaged storage. Therefore, our objective was to determine the effect of extending postmortem aging from 21 to 84 days on consumer eating quality. We hypothesize that tenderness will continue to improve throughout postmortem aging, but flavor may be negatively impacted as the potential for lipid oxidation and off-flavor development increases. Due to the strong reliance of flavor liking on overall liking, we believe overall liking or acceptance may also be negatively impacted at the longest aging periods.

## 2. Materials and Methods

### 2.1. Animals

A total of 108 carcasses were utilized in the current study. All cattle were Continental crossbred steers that were considered grain fed and were sourced from a single supplier. Hormone growth promotants (HGPs) were administered 160 days before harvest to all cattle in this study; specifically, all cattle were implanted with Revalor 200 (Merck Animal Health, DeSoto, KS, USA), which contains 200 mg trenbolone acetate and 20 mg estradiol, following manufacturer recommendations for administration.

### 2.2. Slaughter Procedure, Carcass Grading, and Subprimal Collection

Cattle were slaughtered on a single day at a commercial abattoir in Schuyler, NE. After the carcass chilling period, trained personnel collected carcass data including: marbling score, ossification using USDA skeletal maturity standards [7], 12th rib fat thickness, ribeye area, and HCW. In addition, ultimate pH was collected at the time of carcass grading using a handheld temperature-pH meter equipped with an intermediate junction pH sensor (TPS Model WP-90 with pH sensor part #111227, TPS Pty Ltd., Brendale, QLD, Australia).

Strip loins (Institutional Meat Purchase Specification #180) were collected from the right side of each carcass. Subprimals were vacuum packaged individually and were transported under refrigeration to Texas Tech University, Lubbock, TX. Upon arrival, subprimals were held in chilled storage at 2 °C until 21 days postmortem. Strip loins were fabricated in accordance with MSA protocols [8]. All external fat and connective tissue were removed from strip loins prior to steak fabrication. In addition, the *gluteus medius* was removed from the strip loin leaving only the *longissimus lumborum*. *Longissimus* muscles were fabricated into 2.5 cm steaks and were further processed into smaller pieces measuring approximately 5 × 5 cm. Steak pieces were wrapped in plastic and vacuum packaged as sets of five based on position within the strip loin. Four sets (4 anatomical positions–Anterior [A]1, Anterior [A]2, Posterior [P]3, Posterior [P]4) of five steak pieces were retained from each strip loin for subsequent consumer testing. One of ten postmortem aging periods (every 7 days from 21 days to 84 days) was assigned and balanced within each position to avoid any positional effect when examining postmortem aging. All consumer steaks were vacuum packaged, boxed, maintained in chilled storage (2.0 °C ± 1.0 °C), and frozen on their respective day based on a predetermined postmortem aging designation. Samples were stored at −20 °C until being thawed for consumer sensory testing.

### 2.3. Consumer Sensory Evaluation

Consumer testing was conducted according to MSA grill protocols [8]. Steak samples were thawed at 2–4 °C for 24 h prior to consumer evaluation. All steaks were cooked on a Silex clamshell grill (Model S-143K, Silex Grills Australia Pty Ltd., Marrickville, Australia) with a temperature set at 135 and 142 °C for the top and bottom plate, respectively. The Silex grill was preheated 30 min prior to the start of the panels. A strict and detailed time schedule was followed to ensure all steaks were prepared identically [9]. Each cooking round consisted of ten samples that were cooked at the same time on one grill. All steaks were cooked for 5 min and 45 s, followed by a 3-min rest period. After the rest period, each steak was cut in half into two equal size pieces and served to two separate predetermined consumer panelists.

The Texas Tech University Institutional Review Board approved procedures for use of human subjects for consumer panel evaluation of meat sensory attributes (IRB#: 2017-598). Consumer panels were conducted in the Texas Tech University Animal and Food Sciences Building. Consumer panelists (n = 1080) were recruited from Lubbock, Texas and the surrounding local communities. Panelists had to be regular red meat eaters aged 18 to 75 years old to be able to participate. Each consumer was monetarily compensated and were only allowed to be participate one time. Each session consisted of 20 people with three sessions being conducted on a given evening. Each session lasted approximately 60 min. Each panelist was seated at a numbered booth and was provided with a ballot, plastic utensils, a toothpick, unsalted crackers, a napkin, an empty cup, a water cup, and a cup with diluted apple juice (10% apple juice and 90% water). Each ballot consisted of a demographic questionnaire, seven sample ballots, and a post panel survey regarding beef purchasing habits. Before beginning each panel, consumers were given verbal instructions by Texas Tech personnel about the ballot and the process of testing samples. Panels were conducted in a large classroom that is equipped with standard fluorescent lighting overhead (i.e., no red filters were used) with tables that were divided into individual consumer booths.

Each consumer evaluated seven samples. One steak sample was included in the cooking order as a warm-up sample for consumers and to provide linkage across all testing nights. The link samples were always served in the first position, followed by six test samples served in predetermined, balanced order representing one of ten postmortem aging periods. A Latin-square design was utilized to balance the order and presentation of the samples, ensuring that each product was presented an equal number of times in the six test positions before and after every other product. Each sample had 10 consumer observations (i.e., five consumer steaks all being cut in half and served to two individuals each). Consumers scored palatability traits tenderness, juiciness, flavor liking, and overall liking on 100 mm line scales verbally anchored at 0 (not tender, not juicy, dislike extremely) and 100 (very tender, very juicy, like extremely). Consumers were asked to rate the overall quality or satisfaction of each sample as “unsatisfactory”, “good everyday quality”, “better than everyday quality”, or “premium quality.” The 10 individual scores for each trait were averaged to generate mean sensory scores for each palatability trait and satisfaction prior to analysis. A composite score (MQ4) was calculated using the following equation: tenderness × 0.3 + juiciness × 0.1 + flavor liking × 0.3 + overall liking × 0.3 [8]. Weightings for tenderness and flavor liking have been adjusted from the original weightings by [8] for a balanced contribution to the MQ4 value. The weightings give an indication of the relative importance of the four sensory attributes (tenderness, juiciness, flavor, overall satisfaction) to the final meat quality score.

### 2.4. Statistical Analysis

Data were analyzed in SAS using PROC GLIMMIX (version 9.4, SAS Inst. Inc., Cary, NC, USA). For consumer sensory analyses, postmortem aging period, position, and their interaction were included as fixed effects. Testing day was included as a random effect. Marbling score was included as a covariate, but was significant (*P* < 0.05) only for juiciness. Treatment least squares means were separated with the PDIFF option of SAS using a significance level of *P* ≤ 0.05. Mean separation tests for all pairwise comparisons were performed using the PDIFF function, which requests that *P*-values for differences of all least squares means be produced. The PROC CORR of SAS was used to assess the relationship between consumer eating quality traits by generating Pearson correlation coefficients. The PROC FREQ of SAS was used to summarize consumer demographic information.

## 3. Results and Discussion

### 3.1. Carcass Traits

All carcasses were graded using USDA grading specifications. Carcass characterization can be found in Table 1. The average marbling score was representative of USDA Select, but ranged from USDA Standard to average Choice. However, very few carcasses (5.6%) had marbling scores representative of Choice carcasses (marbling score ≥ 400). As a result of the variation and the known relationship between eating quality and marbling score in the *longissimus lumborum* [10,11,12], marbling score was tested for inclusion as a covariate in the statistical analysis. As previously mentioned, marbling score was only required in the model for consumer juiciness scores (*P* < 0.05).

### 3.2. Consumer Sensory

Demographic characteristics of participating consumers can be found in Table 2. Almost 70% of the participants were aged 20–49 years old, with a relatively even split between those three 10-year age brackets. Seventy-four percent of the population in Lubbock, TX is less than 50 years old [13,14], which aligns with participants in this study. We also believe this percentage is suitable according to the product studied. Participants were evenly distributed between male and female. Most participants (87.7%) identified with Caucasian/White or Hispanic as their ethnic origin, with a fairly even split between the two distinctions. For census purposes, persons who identify as Hispanic or Latino can identify as any race; however, in the latest census data available for Lubbock, TX, 35% reported themselves as Hispanic or Latino, while 65% reported themselves as not Hispanic or Latino [14]. The most common household size consisted of 2–3 adults, representing 74.2% of participants. Nearly half of the participants had no children living in their household. The level of education with the highest proportion of participants was for “some college/technical school” (39.2%), while high school and college graduates collectively accounted for another 45.5%. Additionally, the majority of consumers ate beef at least twice per week (76.9%). The most preferred degree of doneness was medium-rare, with medium and medium-well contributing another 49.1% collectively.

Consumer sensory outcomes can be found in Table 3. No interactions were detected (*P* > 0.05) between postmortem aging and position. Postmortem aging influenced (*P* < 0.01) juiciness, flavor liking, and overall liking, as well as the composite MQ4 score and satisfaction. Somewhat surprisingly, tenderness was not impacted by postmortem aging (*P* = 0.29); however, this could likely be explained by the minimum aging period in this study of 21 days postmortem, where a large portion of proteolysis has potentially already occurred at that stage [15,16,17]. With the exception of flavor liking, position affected (*P* < 0.01) all palatability traits, as well as the composite MQ4 score and satisfaction.

Juiciness generally decreased as postmortem aging increased, but several adjacent aging periods had similar (*P* > 0.05) juiciness scores. For example, samples aged 21 to 35 days had similar and greater juiciness than samples aged 63 days or longer. However, samples aged 42 and 56 days had similar juiciness as those samples aged 21 to 35 days. Flavor liking declined (*P* < 0.01) very clearly as aging period increased. Consumers generally did not differentiate between adjacent aging periods, and so samples were typically grouped together in two to three week aging bands before consumers would indicate flavor liking had declined. Consumers were not as discriminative against overall liking as they were flavor liking, but similar declining trends can be observed in the scores for those two traits. Samples aged 21 to 42 days were most preferred having greater (*P* < 0.05) overall liking and greater (*P* < 0.05) composite MQ4 scores than samples aged 70 to 84 days postmortem. Samples aged 49 to 63 days were essentially intermediate for overall liking and MQ4 score, but had similarities to samples aged both less than 49 days and greater than 63 days. Finally, when assessing satisfaction, a score below 3 indicates consumers scored the sample as “unsatisfactory”. Despite statistical differences (*P* < 0.05), all samples aged 63 days or less would be classified as “good everyday quality.” Consumers were more (*P* < 0.05) satisfied with samples aged up 35 days than samples aged 63 days or longer.

Gruber et al. [2] showed no improvement in tenderness, via decrease in Warner–Bratzler shear force (WBSF) values, beyond 21 days postmortem for *longissimus dorsi* muscle from upper two-thirds USDA Choice carcasses; however, WBSF values did continue to improve through 28 days postmortem for *longissimus dorsi* muscle from USDA Select carcasses, which more closely aligns with the quality grade of carcasses used in the current study. Those results suggest aging *longissimus* muscle beyond 21 days postmortem was only beneficial in carcasses with less marbling (Select). Hughes et al. [6] showed an improvement in eating quality traits (tenderness, juiciness, flavor and overall liking) of beef *longissimus* from 2 to 12 weeks of postmortem aging, but no further improvement at 20 weeks. Lipid oxidation increased throughout the postmortem storage period to levels slightly above acceptable for rancidity detection at 20 weeks, but MQ4 scores suggested the meat would still be acceptable through 20 weeks of storage as classified by consumers [6]. We would like to point out the storage temperature in that trial was maintained at −1.0 °C ± 0.5 °C [6], which was lower and slightly less variable than the current study. Lepper-Blilie et al. [18] also showed tenderness, according to trained panelists, improved linearly as postmortem aging increased from 14 days to 49 d days; however, there was no statistical improvement in tenderness beyond 21 days, which aligns with the current results. When extending aging from 2 to 63 days postmortem, Colle et al. [4] observed no improvement in consumer tenderness after 14 days postmortem, which again supports the current findings. However, acceptability, juiciness, and flavor liking did not differ between the various postmortem aging periods [4], which contradicts the current findings as flavor liking and overall liking decreased with increasing aging period in the present findings. We believe the discrepancy in results between these two studies could again be related to postmortem storage temperature, as previous work has shown increased storage temperature (0 °C vs. 5.0–5.5 °C) of vacuum packaged beef can negatively affect shelf-life and palatability, especially when aging beef beyond 28 postmortem [19,20]. Colle et al. [4] stored vacuum-packaged muscle sections at 0 °C, which is again lower than the current study. Additionally, lipid oxidation increased with postmortem aging, but the authors did not believe their samples had reached the threshold for lipid oxidation based on TBARS values [4]. All samples, regardless of aging period, had less than 1 mg MDA/kg, explaining why they did not see any differences in consumer flavor scores because lipid oxidation had not reached a detectable level by consumers. Lipid oxidation was not evaluated in the current study.

Although postmortem aging can have a positive influence on meat tenderness [2,5,6], its impacts on beef flavor are inconsistent and less well defined, especially at extended postmortem aging periods. In one instance consumers liked the flavor of beef *longissimus lumborum* aged for 12 weeks more than the flavor of beef aged for 2 weeks but no further improvement to flavor liking scores was observed from aging beef for 20 weeks [6]. According to Brewer and Novakofski [1], postmortem aging up to 21 days had no influence on beef flavor; however, extended postmortem aging can promote the development of undesirable flavor characteristics and reduction of beef flavor intensity [5,18]. Lipid oxidation is limited by endogenous antioxidant mechanisms in living muscle [21], but the effectiveness of these antioxidants declines as postmortem aging time increases [22] which could results in increased lipid oxidation [6]. Since lipid oxidation has been linked with rancid flavor [23,24], this could explain why flavor liking decreased as postmortem aging increased in the current study. However, lipid oxidation was not measured in the current study, so we are only speculating that this could be responsible, in part, for the decline in flavor liking scores as postmortem aging increased.

As seen in Table 3, position also affected palatability traits. With the exception of flavor liking, position affected (*P* < 0.01) all palatability traits, as well as the composite MQ4 score and satisfaction. The two anterior-most portions (A1 and A2) were similar and were more tender (*P* < 0.05) than the two posterior-most portions (P3 and P4), which were also similar (*P* > 0.05). A tenderness gradient exists within the *longissimus* muscle [25,26] from the anterior to posterior ends of the strip loin. Muscle fiber angle is affected by steak position [25], and marbling can also vary depending on the anatomical position within the strip loin [27]. Both factors likely contribute to this tenderness gradient observed in the strip loin. The A2 position was juicier (*P* < 0.05) than the two posterior positions, but the A1 position did not differ (*P* > 0.05) in juiciness from any other position in the strip loin. Overall liking generally decreased from the anterior to posterior end of the *longissimus lumborum* muscle. The composite MQ4 score and satisfaction followed the same trend as tenderness, where the anterior portions received greater scores than the posterior portions. Despite statistical differences (*P* < 0.05), all samples would be classified as “good everyday quality.” However, consumers were more (*P* < 0.05) satisfied with samples from the anterior portions of the *longissimus lumborum* than the posterior portions.

### 3.3. Correlations

To estimate the extent to which eating quality scores are linked to overall liking and satisfaction, correlation coefficients between palatability traits, MQ4, and satisfaction scores were determined (Table 4). Consumer overall liking was associated (r = 0.74; *P* < 0.01) with consumer tenderness and juiciness ratings, but most highly related with flavor liking (r = 0.93). Individual palatability traits were strongly correlated to each other (r ≥ 0.67), indicating that individual improvements of these traits could influence the perception of another trait. MQ4 was highly related (*P* < 0.01) to eating quality scores for tenderness, juiciness, flavor liking, and overall liking, as would be expected given it is a composited score of those traits. Satisfaction was positively linked (*P* < 0.01) to all eating quality traits, especially overall liking, and was highly correlated to MQ4 (*P* < 0.01).

## 4. Conclusions

With the exception of flavor liking, position affected all palatability traits, as well as the composite MQ4 score and satisfaction. Despite statistical differences, all samples would be classified as “good everyday quality” regardless of anatomical position within the strip loin. However, consumers were more satisfied with samples from the anterior portions of the *longissimus lumborum* than the posterior portions, likely as a result of greater tenderness from those samples. Postmortem aging influenced juiciness, flavor liking, and overall liking, as well as the composite MQ4 score and satisfaction, but not tenderness. Samples aged 21 to 42 days were most preferred having greater overall liking and greater MQ4 scores than samples aged 70 to 84 days postmortem. Overall liking was clearly driven by flavor liking, as demonstrated by the strongest relationship of the palatability traits. Despite statistical differences, all samples aged 63 days or less would be classified as “good everyday quality.” Consumers were, however, more satisfied with samples aged up 35 days than samples aged 63 days or longer. These results suggest that *longissimus lumborum* samples should not be wet-aged longer than 63 days to prevent potential negative eating experiences for consumers; however, altering storage conditions, specifically reducing storage temperature, could potentially allow for longer chilled storage without such negative effects on flavor liking. Future research involving measurement of lipid oxidation should be conducted to confirm and help define the negative consumer response to flavor and overall liking with extended postmortem aging.

## Figures and Tables

**Table 1:** Simple Statistics for Carcass Traits of Beef Cattle (*n* = 108).

Item	Mean	Standard Deviation	Minimum	Maximum
HCW, kg	364.8	32.8	285.0	436.6
Fat thickness, mm	13.0	4.7	1.3	29.2
LM area, cm ^2^	94.7	11.8	66.1	125.2
Marbling score ^1^	327.2	47.4	200	560
Ossification ^2^	119.9	18.0	110	250
Ultimate pH	5.61	0.06	5.49	6.08

^1^ Marbling: 200 = traces^00^, 300 = slight^00^, 400 = small^00^, 500 = modest^00^ [7]; ^2^ Ossification: 100 = A^00^, 200 = B^00^ [7].

**Table 2:** Demographic characteristics of consumers (*n* = 1080) who participated in consumer sensory panels at Texas Tech University in Lubbock, TX.

Characteristic	Response	% of Consumers
Age Group	<20	7.2
	20–29	24.2
	30–39	24.9
	40–49	20.3
	50–59	12.6
	>60	10.7
Gender	Male	46.8
	Female	53.1
Occupation	Tradesperson	13.3
	Professional	24.8
	Administration	16.6
	Sales and Service	14.6
	Laborer	8.8
	Homemaker	3.1
	Student	10.0
	Unemployed/Retired	8.9
Ethnic Origin	African-American	10.0
	Asian	0.5
	Caucasian/White	41.3
	Hispanic	46.4
	Native American	0.5
	Other	1.3
Household Size (Adults)	1	13.3
	2	57.2
	3	17.0
	4	8.7
	5+	3.8
Household Size (Children)	0	46.6
	1	14.6
	2	23.1
	3	11.4
	4+	4.2
Annual Household Income	<$20,000	13.8
	$20,000–$50,000	32.1
	$50,001–$75,000	21.3
	$75,001–$100,000	16.6
	>$100,000	16.3
Level of Education	Non-High School Graduate	5.3
	High School Graduate	21.7
	Some College/Technical School	39.2
	College Graduate	23.8
	Post-College Graduate	10.0
Beef Consumption	Daily	10.0
	4–5 times a week	26.2
	2–3 times a week	40.7
	Weekly	15.1
	Every other week	5.1
	Monthly	3.0
Preferred Beef Degree of Doneness	Rare	2.9
	Medium-Rare	32.5
	Medium	24.9
	Medium-Well	24.2
	Well-Done	15.5

**Table 3:** The Effects of postmortem aging and position within the *longissimus lumborum* on eating quality scores as rated by consumers (*n* = 1080).

Postmortem Aging	Tenderness ^1^	Juiciness ^1^	Flavor Liking ^1^	Overall Liking ^1^	MQ4 ^2^	Satisfaction ^3^
21 days	66.1	60.8 ^a^	61.1 ^a^	62.6 ^a^	63.0 ^a^	3.37 ^a^
28 days	67.6	59.8 ^ab^	59.1 ^ab^	61.1 ^a^	62.3 ^ab^	3.32 ^ab^
35 days	66.5	60.8 ^a^	59.1 ^ab^	60.0 ^ab^	61.8 ^ab^	3.30 ^ab^
42 days	66.0	58.0 ^abc^	57.0 ^bc^	59.3 ^abc^	60.5 ^abc^	3.18 ^bc^
49 days	65.7	53.6 ^d^	53.4 ^cd^	55.8 ^bcd^	57.9 ^cd^	3.15 ^c^
56 days	65.4	58.3 ^abc^	53.6 ^cd^	56.4 ^bc^	58.5 ^bcd^	3.21 ^bc^
63 days	64.5	55.6 ^cd^	51.7 ^de^	55.0 ^cd^	56.9 ^cd^	3.10 ^cd^
70 days	62.1	53.8 ^d^	46.4 ^fg^	50.2 ^de^	53.0 ^e^	2.97 ^de^
77 days	65.2	55.2 ^cd^	48.3 ^ef^	51.9 ^e^	55.0 ^de^	3.07 ^cde^
84 days	63.3	56.2 ^bcd^	43.8 ^g^	48.5 ^e^	52.2 ^e^	2.94 ^e^
SEM ^4^	1.9	1.8	2.0	2.0	1.8	0.07
*P*-value	0.29	<0.01	<0.01	<0.01	<0.01	<0.01
**Position ^5^**						
A1	69.0 ^a^	58.6 ^ab^	54.1	57.3 ^a^	60.0 ^a^	3.22 ^a^
A2	67.2 ^a^	60.1 ^a^	54.7	58.2 ^ab^	60.0 ^a^	3.24 ^a^
P3	62.8 ^b^	56.7 ^b^	52.4	55.2 ^bc^	56.7 ^b^	3.13 ^b^
P4	62.0 ^b^	53.4 ^bc^	52.1	53.6 ^c^	55.6 ^b^	3.06 ^b^
SEM ^4^	1.5	1.4	1.5	1.6	1.4	0.05
*P*-value	<0.01	<0.01	0.14	<0.01	<0.01	<0.01

^1^ 0 mm = not tender, not juicy, dislike flavor extremely, dislike overall extremely; 100 mm = very tender, very juicy, like flavor extremely, like overall extremely; ^2^ MQ4 = tenderness × 0.3 + juiciness × 0.1 + flavor liking × 0.3 + overall liking × 0.3; ^3^ 2 = unsatisfactory, 3 = good everyday quality, 4 = better than everyday quality, 5 = premium quality; ^4^ Pooled (largest) SEM reported of least sqaures means; ^5^ A = anterior; P = posterior; ^a–g^ Within a column and main effect, least squares means without a common superscript differ (*P* < 0.05).

**Table 4:** Pearson’s correlation coefficients for the relationships between consumer sensory scores of *longissimus lumborum* aged 21 to 84 days postmortem.

Trait	Juiciness	Flavor Liking	Overall Liking	MQ4	Satisfaction
Tenderness	0.73 *	0.67 *	0.74 *	0.86 *	0.71 *
Juiciness		0.68 *	0.74 *	0.81 *	0.71 *
Flavor Liking			0.93 *	0.94 *	0.86 *
Overall Liking				0.96 *	0.88 *
MQ4					0.89 *

* Correlation coefficients were significant (*P* < 0.01).
